# Effect of Chloramine Disinfection of Community Water System on Legionnaires’ Disease Outbreak, Minnesota, USA, 2024

**DOI:** 10.3201/eid3201.251232

**Published:** 2026-01

**Authors:** Molly E. Bledsoe, Apoorva Goel, Maya Adelgren, Timothy M. LaPara, Raymond M. Hozalski

**Affiliations:** University of Minnesota–Twin Cities, Minneapolis, Minnesota, USA (M.E. Bledsoe, A. Goel, M. Adelgren, T.M. LaPara, R.M. Hozalski); Biotechnology Institute, University of Minnesota–Twin Cities, St. Paul, Minnesota, USA (T.M. LaPara, R.M. Hozalski)

**Keywords:** Legionella pneumophila, bacteria, Legionellosis, chloramine, disinfection, water supply, Minnesota, United States

## Abstract

The Minnesota Department of Health identified an outbreak of Legionnaires’ disease in a city in northern Minnesota, USA, in April 2023 that continued until chloramine disinfection of the community water system was implemented. Before chloramine disinfection was implemented, *Legionella pneumophila* was detected in 1 of 16 samples from the drinking water distribution system and in 5 of 10 premise plumbing samples using both cultivation-dependent (Legiolert) and cultivation-independent (digital PCR) assays in this independent investigation. Approximately 11 weeks after disinfection was implemented, all distribution system samples tested negative; however, 1 of 6 Legiolert-tested and 3 of 6 digital PCR–tested premise plumbing samples were positive. After 24 weeks of disinfection, all samples collected from the distribution system and premise plumbing tested negative. Our results show that a community water system supplied by groundwater supported substantial growth of *L. pneumophila* in premise plumbing and that chloramine disinfection halted the outbreak.

Legionnaires’ disease, a severe pneumonia caused by *Legionella pneumophila* bacteria, is an increasingly common disease caused by waterborne pathogens in the United States and other developed countries (*1*–*5*). *L. pneumophila* occurs naturally in surface waters and soils and is commonly found in various engineered water system components, including cooling towers, water distribution systems, showerheads, spas, hot tubs, and humidifiers (*6*,*7*). The primary mode of *L. pneumophila* exposure is through inhalation of contaminated aerosols (*8*).

Although Legionnaires’ disease outbreaks are typically associated with local sources of contamination, a few researchers have attributed Legionnaires’ disease outbreaks to entire community water systems. For example, an outbreak of Legionnaires’ disease occurred concomitantly with the catastrophic lead corrosion event in Flint, Michigan, USA, in 2014 (*9*); however, such associations of outbreaks with entire water systems are rare (*10*). To limit exposure to *L. pneumophila* via building water systems, multiple approaches have been suggested, including the maintenance of a residual disinfectant (*11*–*14*), flushing of infrequently used plumbing systems to minimize stagnation (*15*,*16*), and the maintenance of germicidal temperatures in residential and institutional water heaters (*17*,*18*). Reducing the availability of assimilable organic carbon (AOC) is another strategy to minimize overall growth of bacteria and opportunistic pathogens (*19*).

Beginning in April 2023, a city in northern Minnesota, USA, had 1–2 confirmed cases of Legionnaires’ disease reported each month for 7 consecutive months. After an investigation, the Minnesota Department of Health (MDH) subsequently announced in February 2024 that the community water system was the only common source of exposure among the reported cases, which had continued to mount into 2024. Because the groundwater-supplied system routinely tested negative for total coliforms, that community water system was not required to disinfect its water in accordance with the Ground Water Rule (*20*). In response to the outbreak, the affected utility implemented chloramine disinfection to reduce or eliminate Legionnaires’ disease in the community. We report on the results of an independent investigation in which we collected water samples from the drinking water distribution system and premise plumbing before and after the implementation of chloramine disinfection and analyzed the samples for *L. pneumophila* and other microorganisms of concern (i.e., *Legionella* spp., *Acanthamoeba* spp., *Vermamoeba vermiformis*).

## Materials and Methods

### Study Site 

The community water system is located in northern Minnesota and serves a population of >10,000 persons. Water is withdrawn from 2 aquifers via 5 groundwater wells, supplying as much as 2.25 million gallons/day. The water treatment process includes aeration and filtration for iron and manganese removal, fluoride addition, and softening. The drinking water distribution system comprises ≈81 miles of distribution mains servicing 10.6 square miles; estimated maximum residence time in the system (i.e., water age) is 2–3 days.

### Sample Collection

We collected water samples on 2 occasions before the implementation of chloramine disinfection (February 2024 and May 2024) and 2 occasions after the implementation of chloramine disinfection (September 2024 and December 2024). We collected the samples at the water treatment facility (i.e., raw water and finished water) and from multiple locations throughout the distribution system (Figure; Appendix Table) to provide thorough geographic coverage of the system and a gradient of distances from the water utility. We selected sampling locations, consisting primarily of accessible public buildings, on the basis of recommendations from MDH and utility personnel. No samples were collected from residential buildings. We collected distribution system samples (n = 27) from either a hydrant (n = 4) or from inside buildings at the tap closest to where the service line entered the building (n = 23). We collected additional premise plumbing water samples (cold, n = 11; hot, n = 11) in 3 large institutional buildings from kitchen faucets, utility faucets, or showers. One of those institutional buildings (location B) had a substantial decline in occupancy and water use around the time that chloramine disinfection was implemented. Another one of the institutional buildings (location C) implemented a remediation strategy before the initiation of this study. Location E was included after the first sample collection event. We performed sample collection and analyses for this study independent of other sample collection and analyses done by MDH and utility personnel.

**Figure:**
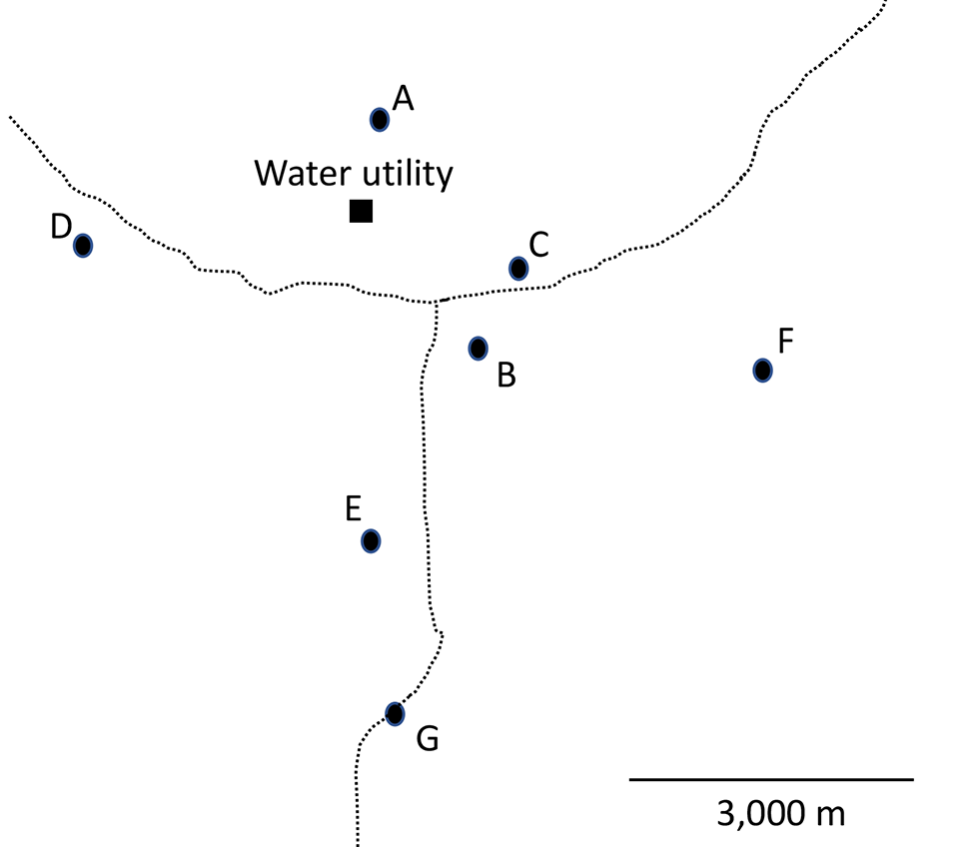
Approximate locations of the sites from which water samples were collected in study of the effect of chloramine disinfection of community water system on Legionnaires’ disease outbreak, Minnesota, USA. Circles indicate community sampling sites A–G. Square indicates water utility. Dotted lines represent 2 major roads that pass through the community.

For water sample collection, we flushed water for 5–10 minutes until it reached a constant temperature. Then, we collected water samples (≈1 L) for digital PCR in autoclaved polypropylene bottles containing sodium thiosulfate to quench any residual disinfectant. Similarly, we collected 100-mL samples in manufacturer-provided sterile bottles containing sodium thiosulfate for culture-based analyses of *L. pneumophila* and total coliforms. We collected samples for total organic carbon and AOC, a measure of the organic carbon readily available for assimilation by bacteria, in carbon-free glass bottles. We immediately placed all water samples on ice for same-day transport back to the laboratory and processed all samples within 48 hours of collection.

### Water Quality Analyses

We measured temperature, pH, and chlorine concentrations onsite immediately before water sample collection. We measured temperature and pH using a handheld meter (PH60 pH tester; Apera Instruments, https://aperainst.com). We determined total chlorine using the N,N-diethyl-p-phenylenediamine method and a portable colorimeter (DR3000 or SL1000; Hach, https://www.hach.com) according to the manufacturer’s protocol. We determined total organic carbon concentrations using a TOC analyzer (TOC-L series; Shimadzu, https://www.shimadzu.com) calibrated using potassium hydrogen phthalate standards. We measured AOC by inoculating pasteurized water samples with 2 strains of bacteria obtained from the American Type Culture Collection, *Pseudomonas fluorescens* strain P17 (ATCC 49642) and *Spirillum* sp. strain NOX (ATCC 49643), incubating them at room temperature, and enumerating the organisms over time via plating as described previously (*21*).

### Culture-Based Enumeration of Microorganisms

We enumerated *L. pneumophila* via cultivation using the Legiolert method and total coliforms by the Colilert method (IDEXX Laboratories, https://www.idexx.com), in accordance with the manufacturer’s instructions. We performed quality assurance and quality control of the Legiolert assay using samples of *L. pneumophila* (ATCC 33156; positive control) and *Enterococcus faecalis* (ATCC 29212; negative control). We extracted DNA from >1 positive well from every sample positive by the Legiolert method and subjected it to digital PCR, targeting the *mip* and *wzm* genes to validate the results.

### Sample Processing and DNA Extraction

We concentrated microorganisms from each water sample (≈1 L) onto a mixed cellulose ester filter 47 mm in diameter with nominal pore size of 0.2 µm (MilliporeSigma, https://www.sigmaaldrich.com) via vacuum filtration. We prepared blank (control) filters (n = 15) by filtering 2 mL of autoclaved tap water through a clean filter. We immediately placed each filter into a PowerWater Bead Pro Tube (QIAGEN, https://www.qiagen.com) containing PW1 lysis buffer and stored them at −20°C. We then extracted DNA from the filter membranes and from biomass removed from positive Legiolert wells via syringe, using the DNeasy PowerWater Kit and a QIAcube Connect system (QIAGEN) according to the manufacturer’s protocol. All blank (control) filters were negative for all PCR targets except for 16S rRNA genes.

### Quantitative PCR

We performed real-time quantitative PCR to quantify total bacteria (i.e., 16S rRNA genes) (*22*) and *Vermamoeba vermiformis* (i.e., 18S rRNA genes) (*23*) using a CFX Connect Real-Time System (Bio-Rad Laboratories, https://www.bio-rad.com). We determined the quantities of unknown samples against calibration curves prepared with known quantities of synthetic gBlock gene fragments (Integrated DNA Technologies, https://www.idtdna.com). The limit of detection (LOD) for all bacteria was 10^5.0^ gene copies/L; the LOD for *V. vermiformis* was 10^3.0^ gene copies/L. We used digital PCR to quantify *Legionella* spp. (*ssrA*), *L. pneumophila* (*mip*), *L. pneumophila* serogroup 1 (*wzm*), and *Acanthamoeba* (18S rRNA for *Acanthamoeba*) (*24*,*25*). Each digital PCR involved multiplexing 4 target genes using the QIAcuity One on the QIAcuity Nanoplate 8.5K 96-well (QIAGEN) following manufacturer’s standard operational procedures. The LOD for the digital PCRs was 10^2.34^ gene copies/L based on a minimum requirement of 3 positive partitions for an assay to be designated as a valid detection (Appendix Tables 7, 8). 

We compared pairwise concentrations of microorganisms by Wilcoxon rank sum test. Differences of p<0.05 were statistically significant.

## Results

### Water Quality Characteristics

We tested all water samples collected from the distribution system (Appendix Tables 2–6); all tested negative for total coliforms (Appendix Tables 2–5). Typical temperatures of the distribution system samples were 4.4°C–15.5°C, except for the September samples that had higher temperatures (11.3°C–20.9°C) (Appendix Tables 2–5). Among the premise plumbing samples, hot water temperatures ranged from 21.4°C (water heater was not in use at the time of sample collection) to 47.2°C (Appendix Tables 2–5). Similarly, cold water temperatures typically ranged from 9.4°C to 15.5°C, except for the September samples, for which temperature range was 19.7°C–21.7°C. Water sample pH range was 7.2–8.4; median pH was 8.1.

We assumed negligible total chlorine concentrations and did not measure levels in February 2024 and May 2024 before disinfection was initiated. Total chlorine concentration in the distribution system in September 2024 was 0.3–1.8 mg/L as Cl_2_ and in December 2024 was 1.2–2.4 mg/L as Cl_2_. Total chlorine concentrations in the cold-water premise plumbing samples were similar to the distribution system samples. In contrast, the total chlorine concentrations in the hot-water samples were lower (0.1–0.8 mg/L as Cl_2_).

Total organic carbon concentrations were consistent from the source water through the distribution system (mean +SD = 2.2 +0.4 mg/L) (Appendix Table 6). In contrast, AOC concentrations varied substantially depending on sample location and date. AOC concentrations in the source water collected from 5 different wells (before treatment) and in the finished water (after treatment) were similar; the range was 2.2–37.5 µg/L. In December 2024, AOC concentrations in the distribution system were similar to the well water and finished water (8.8–20.3 µg/L). In contrast, in September 2024, AOC concentrations increased from 37.1 µg/L in the finished water to 117–157 µg/L in the distribution system.

### Quantification of Total Bacteria in Distribution System and in Premise Plumbing Water

We observed significantly lower concentrations of bacteria (Wilcoxon p<10^−5^) in the distribution system and premise plumbing samples after introduction of chloramines (Appendix Tables 9–12). Before disinfection, we quantified substantial concentrations of bacteria in both distribution system samples (10^5.9^–10^7.7^ gene copies/L; median 10^7.5^ gene copies/L) and in premise plumbing samples (10^7.1^–10^9.5^ gene copies/L; median 10^7.5^ gene copies/L). After disinfection, we observed substantial decreases in the concentrations of bacteria in the distribution system samples (10^5.2^–10^7.3^ gene copies/L; median 10^5.7^ gene copies/L) and in the premise plumbing samples (10^5.0^–10^8.8^ gene copies/L; median 10^6.1^ gene copies/L).

### Quantification of *V. vermiformis* and *Acanthamoeba* spp.

All distribution system and premise plumbing samples were negative for *Acanthamoeba* organisms (n = 53). In contrast, we detected *V. vermiformis* frequently in both the distribution system and premise plumbing samples. Before disinfection, the frequency of detection (FOD) in the distribution system samples was 100% (n = 16), corresponding to concentrations of 10^3.0^–10^4.7^ gene copies/L (median 10^4.2^ gene copies/L). After chloramine disinfection was implemented, the FOD decreased to 62.5% (10/16 samples), with corresponding significantly lower concentrations of LOD to 10^5.0^ gene copies/L (median 10^3.0^ gene copies/L) (Wilcoxon p = 0.003). Similarly, the FOD in the premise plumbing samples was 100% (n = 10) before disinfection but decreased to 58.3% (7/12 samples) after chloramine disinfection was implemented.

### Quantification of *L. pneumophila* via Legiolert

Before the use of chloramine disinfection, *L. pneumophila* was rarely detected in the distribution system but was frequently detected in premise plumbing samples via Legiolert (Table 1). We detected *L. pneumophila* in 1 distribution system sample before disinfection (1/16 samples) at the LOD (10^1.0^ most probable number [MPN]/L). In contrast, half (5/10 samples) of the premise plumbing samples before disinfection were positive by the Legiolert assay; most of the Legiolert-negative results came from location C, which had performed a remediation procedure to prevent additional cases of Legionnaires’ disease before we began our study. The 3 positive hot water samples had *L. pneumophila* concentrations of 10^2.4^–10^4.0^ MPN/L; the corresponding cold water samples had concentrations ranging from below the LOD to 10^3.7^ MPN/L.

**Table 1:** Concentrations of *Legionella pneumophila* as determined by Legiolert assay in water samples collected in study of *Legionella* and chloramine disinfection within a community water system, Minnesota, USA*

Location	Description	Concentration, log_10_ MPN/L			
		2024 Feb	2024 May	2024 Sep	2024 Dec
Water utility	Finished water	<LOD	<LOD	<LOD	<LOD
A	Distribution system	1	<LOD	<LOD	<LOD
B	Distribution system	<LOD	<LOD	<LOD	<LOD
	Premise, cold	3.7	2.0	<LOD	<LOD
	Premise, hot	3.7	2.4	1.6	<LOD
C	Distribution system	<LOD	<LOD	<LOD	<LOD
	Premise, cold	<LOD	<LOD	<LOD	<LOD
	Premise, hot	<LOD	<LOD	<LOD	<LOD
D	Distribution system	<LOD	<LOD	<LOD	<LOD
E	Distribution system	ND	<LOD	<LOD	<LOD
	Premise, cold	ND	<LOD	<LOD	<LOD
	Premise, hot	ND	4.0	<LOD	<LOD
F	Distribution system	<LOD	<LOD	<LOD	<LOD
G	Distribution system	<LOD	<LOD	<LOD	<LOD

*Samples were collected on different dates and from different locations. Samples were classified as being from the distribution system or from the premise plumbing (cold or hot). Chloramine disinfection was initiated in June 2024. LOD, limit of detection; MPN, most probable number; ND, not determined because no sample was collected.

After disinfection was implemented, we noted a substantial decrease in the FOD and in the concentrations of *L. pneumophila* determined via Legiolert. We did not detect *L. pneumophila* in any of the distribution system samples (n = 16) and in only 1/12 premise plumbing samples. That positive sample, which was collected from the hot water at location B in September 2024, had an *L. pneumophila* concentration of 10^1.6^ MPN/L.

### Quantification of *Legionella* by Digital PCR

We detected *Legionella* spp. (target gene *ssrA*) by digital PCR in numerous distribution system and premise plumbing samples (Appendix Tables 9–12). Before disinfection, the concentrations of *Legionella* spp. in the distribution system samples (range from below the LOD to 10^4.2^ gene copies/L) were slightly lower than those in premise plumbing (range 10^2.6^–10^4.7^ gene copies/L). A significant (Wilcoxon p<0.001) decrease in the concentration of *Legionella* spp. in the distribution system samples (range from below the LOD to 10^3.4^ gene copies/L) and in the premise plumbing samples (range from below the LOD to 10^4.8^ gene copies/L, with 1 outlier at 10^8.2^ gene copies/L) occurred after implementation of chloramine disinfection.

We quantified the concentrations of both *L. pneumophila* (target gene *mip*) and *L. pneumophila* serogroup 1 (target gene *wzm*) in distribution system and premise plumbing samples (Appendix Tables 9–12). Because the results for those 2 assays were very similar, we describe here only the results for *L. pneumophila* (target gene *mip*). We did not detect *L. pneumophila* by digital PCR in any distribution system samples either before or after the implementation of disinfection (Table 2). Before disinfection, however, we detected *L. pneumophila* in several premise plumbing samples (Table 2). The FOD of *L. pneumophila* was greater for hot water samples (80%; 4/5 samples) than for cold water samples (20%; 1/5 samples) with concentrations in hot water samples ranging from below the LOD to 10^4.3^ gene copies/L and in cold water samples from below the LOD to 10^3.2^ gene copies/L. We collected most of the PCR-negative samples from location C, which had undergone a building remediation in response to the Legionnaires’ disease outbreak before our sample collection campaign. 

**Table 2:** Concentrations of *Legionella pneumophila* as determined by digital PCR targeting *mip* genes in water samples collected in study of *Legionella* and chloramine disinfection within a community water system, Minnesota, USA*

Location	Description	Concentration, log_10_ gene copies/L			
		2024 Feb	2024 May	2024 Sept	2024 Dec
Water utility	Finished water	<LOD	<LOD	<LOD	<LOD
A	Distribution system	<LOD	<LOD	<LOD	<LOD
B	Distribution system	<LOD	<LOD	<LOD	<LOD
	Premise, cold	3.2	<LOD	3.9	<LOD
	Premise, hot	4.3	3.1	8.2	<LOD
C	Distribution system	<LOD	<LOD	<LOD	<LOD
	Premise, cold	<LOD	<LOD	<LOD	<LOD
	Premise, hot	2.5	<LOD	<LOD	<LOD
D	Distribution system	<LOD	<LOD	<LOD	<LOD
E	Distribution system	ND	<LOD	<LOD	<LOD
	Premise, cold	ND	<LOD	<LOD	<LOD
	Premise, hot	ND	3.9	2.6	NM
F	Distribution system	<LOD	<LOD	<LOD	<LOD
G	Distribution system	<LOD	<LOD	<LOD	<LOD

*Samples were collected on different dates and from different locations. Samples were classified as being from the distribution system or from the premise plumbing (cold or hot). Chloramine disinfection was initiated in June 2024. LOD, limit of detection; ND, not determined because no sample was collected; NM, not measured because the sample was lost during processing.

After disinfection, the FODs and concentrations of *L. pneumophila* in premise plumbing samples decreased substantially. *L. pneumophila* was detected in 2/6 premise plumbing samples collected in September 2024 but not in any sample (n = 6) collected in December 2024. One of the samples collected in September 2024 had the highest observed concentration of *L. pneumophila* (10^8.2^ gene copies/L); that sample was collected at the same time and location as the lone Legiolert-positive sample after chloramine disinfection had been implemented.

## Discussion

Most Legionnaires’ disease outbreaks are attributed to a specific, localized issue such as water stagnation in a large building or an improperly maintained cooling tower (*6*,*7*). In February 2024, however, MDH publicly announced that the community water supply was the likely source of *L. pneumophila* connected to an outbreak of Legionnaires’ disease that eventually comprised 34 confirmed cases (Appendix Figure), including 2 deaths and 30 hospitalizations. After the announcement, personnel from MDH, which is also responsible for regulating drinking water quality in the state of Minnesota, asked us to perform an independent, complementary analysis of the community water system. Our initial analysis identified *L. pneumophila* in multiple institutional buildings throughout the community water system at concentrations sufficiently high (>1,000 MPN or gene copies/L) to require corrective action (*12*). Our subsequent analysis, performed after the implementation of chloramine disinfection in June 2024, revealed less frequent detections and lower concentrations of *L. pneumophila*. In addition, there were no additional cases of Legionnaires’ disease after the implementation of chloramine disinfection. Our results, therefore, demonstrate that the community water system was susceptible to growing substantial concentrations of *L. pneumophila* within premise plumbing, contributing to an outbreak of Legionnaires’ disease, and that chloramine disinfection effectively halted the outbreak.

To our knowledge, researchers have previously identified community water systems in the United States as the principal factor in outbreaks of Legionnaires’ disease on 3 previous occasions. One of those was associated with the water crisis in Flint, Michigan (*9*), and the other 2 were attributed to a failure to maintain a strong residual disinfectant in community water systems supplied by surface water (*26*,*27*). The Minnesota outbreak of Legionnaires’ disease, therefore, was unique because it was triggered by a community water system supplied by groundwater. The source of the water supply is particularly pertinent because in the United States, community water systems supplied by groundwater that routinely test negative for total coliforms are not required to disinfect the water before distribution or maintain a residual disinfectant throughout the distribution system (*20*).

We suspect that an unexpectedly high AOC content in the groundwater supply was a factor contributing to this outbreak of Legionnaires’ disease. It is believed that *L. pneumophila* growth in water distribution systems primarily occurs inside amoeba (e.g., *V. vermiformis*) that graze on biofilms that form on the interior surfaces of water distribution and premise plumbing rather than in the drinking water directly (*28*). Thus, the AOC in water supplies could indirectly contribute to *L. pneumophila* proliferation by enabling the growth of biofilms on distribution system mains and premise plumbing piping (*19*). Although ground water typically has low concentrations of bioavailable organic carbon (*21*,*29*–*31*), AOC levels in the drinking water in our study routinely exceeded the suggested threshold for microbiologically stable water in the absence of a residual disinfectant of 10–20 µg/L (*21*) and at times exceeded the threshold for water containing a residual disinfectant of 50–100 µg/L (*32*). Of note, AOC concentrations in the distribution system often were greater than those in the raw groundwater and finished water; identifying the cause of this increase within the distribution system warrants further investigation.

From a practical perspective, our study demonstrates that maintaining a residual disinfectant throughout the distribution system is a robust approach to suppress the growth of *Legionella* spp. bacteria (as *ssrA* genes), *L. pneumophila* bacteria (via Legiolert and digital PCR targeting *mip* and *wzm* genes), other bacteria (as 16S rRNA genes), and amoebas (especially *V. vermiformis*) (*28*,*33*). Another theory is that the lack of residual disinfectant was a pertinent factor contributing to this outbreak of Legionnaires’ disease. Although most public water systems in the United States practice primary disinfection (i.e., as part of the treatment process before distribution) and maintain a residual disinfectant, the Ground Water Rule (*20*) enables community water systems that test negative for total coliforms to forego disinfection. As of December 2025, there are >250 community water systems in Minnesota that do not practice disinfection, including 10 such systems that serve populations of >1,000 persons.

In conclusion, our study provides evidence that a community water system was the source of a Legionnaires’ disease outbreak that was subsequently resolved by implementing chloramine as a residual disinfectant throughout the drinking water distribution system. The principal water quality factor leading to the outbreak was a lack of residual disinfectant in the drinking water distribution system. Perhaps more important, however, we conjecture that unexpectedly high concentrations of AOC contributed to the overall growth of bacteria and the occurrence of *L. pneumophila*. Although groundwater is generally low in AOC (<50 µg/L [*34*]), that water quality parameter is rarely monitored in the United States. We advocate, therefore, for AOC testing to identify community water systems at greater risk for bacterial and possibly *L. pneumophila* growth, especially those supplied by ground water that do not use disinfection and any disinfected system that struggles to maintain a residual throughout their distribution system. Furthermore, nondisinfected ground water systems and disinfected systems that struggle to maintain a residual also should consider periodic monitoring for *L. pneumophila* and disinfection or disinfection boosting when positive samples or cases of Legionnaires’ disease are encountered.

Appendix Additional information from study of the effect of chloramine disinfection of community water system on Legionnaires’ disease outbreak, Minnesota, USA.
