# Supplementary material for: Effect of Chloramine Disinfection of Community Water System on Legionnaires’ Disease Outbreak, Minnesota, USA, 2024
Source: Emerg Infect Dis. 2026 Jan;32(1):74–81. doi: 10.3201/eid3201.251232 (PMC12869989; doi:10.3201/eid3201.251232)
Supplement: Appendix — Additional information from study of the effect of chloramine disinfection of community water system on Legionnaires’ disease outbreak, Minnesota, USA. [file 25-1232-Techapp-s1.pdf]

Article DOI: https://doi.org/10.3201/eid3201.251232

Effect of Chloramine Disinfection of Community Water System on Legionnaires' Disease Outbreak, Minnesota, USA, 2024

Appendix

Quantitative PCR

Real time PCR was used to quantify all bacteria and *Vermamoeba vermiformis* using a CFX Connect Real-Time System (Bio-Rad Laboratories; Hercules, California). The PCR mastermix included 1× SsoAdvanced Universal SYBR Green Supermix (Bio-Rad), DNase-free water, 20 µg of bovine serum albumin, and forward (concentration = 0.5 µM) and reverse primers (concentration = 0.25 µM); the total volume of each real time PCR assay was 20 µL. The thermal cycle for these real time PCR assays included an initial denaturation at 95°C for two minutes followed by 40 cycles of denaturation (95°C) and anneal/extend (60°C for Bacteria; 56°C for *Vermamoeba vermiformis*). The concentrations of unknown samples were computed based on a comparison to a standard curve of known concentrations of gBlock standards (Integrated DNA Technologies; Coralville, Iowa). Amplification curves of unknown samples were visually compared to the amplification curves of gBlock standards to determine if the unknown samples inhibited PCR; no PCR inhibition was observed for any sample analyzed in this study.

Digital PCR was used to quantify *Legionella* spp., *L. pneumophila*, and *L. pneumophila* serogroup (SG) 1 (i.e., gene targets *ssrA*, *mip*, and *wzm*, respectively) and *Acanthamoeba* (18S rRNA for *Acanthamoeba*). All primers and probes were synthesized by Integrated DNA Technologies, Inc. (Skokie, Illinois). All digital PCR assays included multiplexing four target genes using the QIAcuity One instrument and a QIAcuity Nanoplate 8.5K 96-well. (Qiagen; Hilden, Germany) following manufacturer's instructions. The PCR mastermix included 1× Qiagen digital PCR solution, 12 µg of bovine serum albumin, 0.6 U of *EcoR1* restriction enzyme, 6 µL of DNA extract, DNase-free water, and primers and probes for *ssrA* (primer

concentrations = 0.5 μM; probe concentration = 0.1 μM), *mip* (primer concentrations = 0.5 μM; probe concentration = 0.1 μM), *wzm* (primer concentrations = 0.5 μM; probe concentration = 0.1 μM), and *Acanthamoeba* (primer concentrations = 0.2 μM; probe concentration = 0.1 μM). Most probes were PrimeTime qPCR Probes (IDT); the exception was the probe that targeted *ssrA*, which was an MGB Eclipse Probe (IDT). Probes were tagged with a specific fluorophore (HEX for *ssrA*, ATTO 550 for *mip*, 6-FAM for *wzm*, and Texas Red for 18S rRNA genes/*Acanthamoeba*). The total reaction mixture had a volume of 12 μL. The thermal cycle involved an initial denaturation at 95°C for two minutes followed by 55 cycles of denaturation (95°C) and annealing (60°C).

Each digital PCR assay included positive controls (comprised of either gBlocks or previously-identified positive samples), no-template controls, and filter-blank controls. Once amplification was completed, the endpoint fluorescence was measured and a fluorescence threshold for each target gene was manually set based on the relative results from positive controls and no-template controls using the Qiagen Software Suite (Qiagen; Hilden, Germany). Each partition was then classified as positive if it exceeded the fluorescence threshold. A minimum of three positive partitions was required for a sample to be deemed positive. The limit of detection of each digital PCR assay varied slightly, depending on the volume of water sample filtered (the target volume for filtration was 1.00 L but some sample-to-sample variation occurred) and the number of valid partitions per assay. In general, the detection limit for all digital PCR assays was $10^{2.34 \pm 0.02}$ gene copies per liter. All no-template and blank controls were negative in all digital PCR assays for all target genes. The absolute concentration (copies of target genes per volume of reaction mix) was then calculated via fitting to a Poisson distribution using the Qiagen Software Suite. Primer, probe, and gBlock sequences for all quantitative PCR assays are shown in Appendix Tables 7 and 8.

Appendix Table 1. Descriptions of locations from which water samples were collected.

Location	Description
Water utility	Finished water (i.e., following treatment) collected at the water treatment facility
A	Large institutional building. Only distribution system samples were collected.
B	Large institutional building. Both distribution system (building inlet) and premise plumbing (cold and hot) samples were collected
C	Large institutional building. Both distribution system (building inlet) and premise plumbing (cold and hot) samples were collected
D	Small building owned by the water utility.
E	Large institutional building. Both distribution system (building inlet) and premise plumbing (cold and hot) samples were collected
F	Small institutional building. Only distribution system (building inlet) samples were collected.
G	Flushing hydrant near water tower

Appendix Table 2. Additional water quality parameters quantified from different locations within the community water system in February 2024*

Location	Description	Temperature, °C	pH	Total coliforms, MPN/100 mL	Total chlorine, as Cl_2, mg/L
Water utility	Finished water	10.4	7.6	<LOD	ND
A	Distribution system	8.2	7.6	<LOD	ND
B	Distribution system	8.5	7.6	<LOD	ND
B	Premise, cold	13.2	7.6	ND	ND
B	Premise, hot	42.3	7.5	ND	ND
C	Distribution system	8.6	7.8	<LOD	ND
C	Premise, cold	9.4	7.6	ND	ND
C	Premise, hot	39.1	7.6	ND	ND
D	Distribution system	15.5	7.6	<LOD	ND
E	Distribution system	ND	ND	ND	ND
E	Premise, cold	ND	ND	ND	ND
E	Premise, hot	ND	ND	ND	ND
F	Distribution system	6.2	7.6	<LOD	ND
G	Distribution system	4.4	7.6	ND	ND

*LOD, limit of detection; ND, not determined because no sample was collected.

Appendix Table 3. Additional water quality parameters quantified from different locations within the community water system in May 2024*

Location	Description	Temperature, °C	pH	Total coliforms, MPN/100 mL	Total chlorine as Cl_2, mg/L
Water utility	Finished water	12.0	8.1	<LOD	ND
A	Distribution system	14.4	8.1	<LOD	ND
B	Distribution system	13.0	8.1	<LOD	ND
B	Premise, cold	15.2	8.0	ND	ND
B	Premise, hot	21.4	8.0	ND	ND
C	Distribution system	12.8	8.1	<LOD	ND
C	Premise, cold	13.3	8.1	ND	ND
C	Premise, hot	45.7	8.0	ND	ND
D	Distribution system	8.1	8.0	<LOD	ND
E	Distribution system	9.8	8.1	<LOD	ND
E	Premise, cold	11.8	8.1	ND	ND
E	Premise, hot	28.8	8.0	ND	ND
F	Distribution system	8.2	8.1	<LOD	ND
G	Distribution system	11.5	8.1	<LOD	ND

*LOD, limit of detection; ND, not determined because no sample was collected.

Appendix Table 4. Additional water quality parameters quantified from different locations within the community water system in September 2024*

Location	Description	Temperature, °C	pH	Total coliforms, MPN/100 mL	Total chlorine as Cl_2, mg/L
Water utility	Finished water	11.3	8.2	<LOD	1.5
A	Distribution system	16.5	8.2	<LOD	0.3
B	Distribution system	20.9	7.6	<LOD	0.8
B	Premise, cold	21.7	8.2	ND	1.1
B	Premise, hot	46.4	8.0	ND	0.1
C	Distribution system	15.5	8.2	<LOD	1.8
C	Premise, cold	20.6	8.3	ND	ND
C	Premise, hot	38.6	8.2	ND	ND
D	Distribution system	14.7	8.2	<LOD	1.3
E	Distribution system	18.6	8.2	<LOD	1.0
E	Premise, cold	19.7	8.3	ND	1.1
E	Premise, hot	47.2	7.9	ND	0.2
F	Distribution system	16.9	8.4	<LOD	1.3
G	Distribution system	20.4	8.2	<LOD	1.5

*LOD, limit of detection; ND, not determined because no sample was collected.

Appendix Table 5. Additional water quality parameters quantified from different locations within the community water system in December 2024*

Location	Description	Temperature, °C	pH	Total coliforms, MPN/100 mL	Total chlorine as Cl_2, mg/L
Water utility	Finished water	7.3	8.1	<LOD	2.4
A	Distribution system	13.3	8.3	<LOD	1.9
B	Distribution system	12.8	8.1	<LOD	2.4
B	Premise, cold	14.7	8.2	ND	2.1
B	Premise, hot	44.0	8.0	ND	0.2
C	Distribution system	11.9	8.1	<LOD	2.0
C	Premise, cold	13.0	8.2	ND	1.9
C	Premise, hot	41.6	8.1	ND	0.8
D	Distribution system	11.5	8.1	<LOD	1.2
E	Distribution system	12.1	8.2	<LOD	1.5
E	Premise, cold	10.9	8.2	ND	1.6
E	Premise, hot	29.7	8.0	ND	0.6
F	Distribution system	13.9	8.2	<LOD	1.2
G	Distribution system	8.2	7.2	<LOD	1.7

*LOD, limit of detection; ND, not determined because no sample was collected.

Appendix Table 6. Concentrations of assimilable organic carbon and total organic carbon collected from various locations within the community water system

Sample date	Description	Assimilable organic carbon as C-acetate, µg/L	Total organic carbon, mg/L
July 2024	Municipal well 6	9.3	2.2
	Finished water	2.2	2.3
	Location C	8.8	2.4
	Location G	20.6	2.4
Sept. 2024	Municipal well 1	13.6	2.2
	Municipal well 2	37.5	0.9
	Municipal well 3	20.9	2.3
	Municipal well 4	24.2	2.5
	Municipal well 6	30.6	2.6
	Finished water	37.1	2.2
	Location C	117	2.2
	Location G	157	2.7
Dec. 2024	Municipal well 6	10.3	2.5
	Finished water	26.8	2.0
	Location C	18.6	2.0
	Location G	20.2	2.1

Appendix Table 7. The DNA sequences of all primers and probes used for quantitative PCR as part of this investigation*

Gene target	Primer/probe sequence (5′→3′)
16S rRNA gene for bacteria	F: CCT ACG GGA GGC AGC AG<br>R: ATT ACC GCG GCT GCT GG
18S rRNA gene for *Vermamoeba vermiformis*	F: TTA CGA GGT CAG GAC ATG T<br>R: GAC CAT CCG GAG TTC TCG
ssrA, *Legionella* spp.	F: GGC GAC CTG GCT TC<br>R: GGT CAT CGT TTG CAT TTA TAT TTA<br>P: ACG TGG GTT GCA A
mip, *Legionella pneumophila*	F: TTG TCT TAT AGC ATT GGT GCC G<br>R: CCA ATT GAG CGC CAC TCA TAG<br>P: CGG AAG CAA TGG CTA AAG GCA TGCA
wzm, *Legionella pneumophila* serogroup 1	F: TGC CTC TGG CTT TGC AGT TA<br>R: CAC ACA GGC ACA GCA GAA ACA<br>P: TTT ATT ACT CCA CTC CAG CGA T
18S rRNA gene for *Acanthamoeba*	F: CCC AGA TCG TTT ACC GTG AA<br>R: TAA ATA TTA ATG CCC CCA ACT ATC C<br>P: CCA CCG AAT ACA TTA GCA TGG

*F, forward primer; P, probe; R, reverse primer.

Appendix Table 8. gBlock sequences used as standards (real time PCR) and positive controls (digital PCR) as part of this investigation*

Gene	gBlock sequence (5′→3′)	GenBank accession no.
16S rRNA gene for bacteria	TGAGACACGGTCCAGACTCCTACGGGAGGCAGCAGTGGGGAATATTG<br>CACAATGGGCGCAAGCCTGATGCAGCCATGCCGCGTGTATGAAGAAG<br>GCCTTCGGGTTGTAAAGTACTTTCAGCGGGGAGGAAGGGAGTAAAGT<br>TAATACCTTTGCTCATTGACGTTACCCGCAGAAGAAGCACCGGCTAAC<br>TCCGTGCCAGCAGCCGCGGTAATACGGAGGGTGCAAGC	KR190116.1
18S rRNA gene for *Vermamoeba vermiformis*	TCAACACGGGGAAACTTACGAGGTCAGGACACTGTGAGGATTGACAG<br>ATTGAAAGCTCTTTCTTGATTCAGTGGGTGGTGGTGCATGGCCGTTCT<br>TAGTTGGTGGAGTGATTTGTCTGGTTAATTCCGTTAACGAACGAGACC<br>TTAACCTGCTAAATAGTCACGCGAACCGGTCCGCAAGGGCTCAAAGTT<br>CGCGGCTCGACTTCTTAGAGGGACTATTCGCACCCGCCAGCGAATGG<br>AAGTTTGAGGCAATAACAGGTCTGTGATGCCCTTAGATGTTCTCGGCC<br>GCACGCGCGTTACACTGACGGAGTCAACGAGCGTTTTTCCTTGCCCG<br>ATAGGGCCGGGTAACCTCCTGAAACTCCGTCGTGATGGGGATAGATC<br>ATTGCAATTATTGATCTTGAACGAGGAATTCCTAGTAAGCGCGAGTCAT<br>CAACTCGCGCTGATTACGTCCCTGCCCTTTGTACACACCGCCCGTCGC<br>TCCTACCGATTGAACGGTCCGGCGAGAACTCCGGATGGTCGGCACGC<br>AGGGGTCA	AF426157.1
ssrA, *Legionella* spp.	AGTGTACAGAATGGGGGGCGACCTGGCTTCGACGTGGGTTGCAAAAC<br>CGGAAGTGCATGCCGAGAAGGAGATCTCTCGTAAATAAGACTCAATTA<br>AATATAAATGCAAACGATGAAAACTTTGCTGGTGGGG	CP015927.1
mip, *Legionella pneumophila*	ACAGACAAGGATAAGTTGTCTTATAGCATTGGTGCCGATTTGGGGAAG<br>AATTTTAAAAATCAAGGCATAGATGTTAATCCGGAAGCAATGGCTAAAG<br>GCATGCAAGACGCTATGAGTGGCGCTCAATTGGCTTTAACCGAACAGC	CP015927.1
wzm, *Legionella pneumophila* serogroup 1	ATATTAAACATTTTATGCCTCTGGCTTTGCAGTTATTTTATTACTCCACT<br>CCAGCGATTTACCCTGTTTCTGCTGTGCCTGTGTGGGCTAAACCATGG<br>TA	AJ007311.1
18S rRNA gene for *Acanthamoeba*	GGTGGGTTCCTGGGGCCCAGATCGTTTACCGTGAAAAAATTAGAGTGT<br>TCAAAGCAGGCAGATCCAATTTTCTGCCACCGAATACATTAGCATGGG<br>ATAATGGAATAGGACCCTGTCCTCCTATTTTCAGTTGGTTTTGGCAGC<br>GCGAGGACTAGGGTAATGATTAATAGGGATAGTTGGGGGCATTAATAT<br>TTAATTGTCAGAGGTGAA	MF399030.1

Appendix Table 9. Concentrations of various organisms in water samples collected from different locations within a community water system in February 2024*

Location	All bacteria	*Legionella* spp.	*L. pneumophila*	*L. pneumophila SG1*	*Acathamoeba*	*V. vermiformis*
Finished water	6.9	2.8	<LOD	<LOD	<LOD	3.6
A	7.4	3.6	<LOD	<LOD	<LOD	4.2
B	7.6	2.6	<LOD	<LOD	<LOD	4.6
B, cold	7.1	3.8	3.2	3.1	<LOD	4.0
B, hot	9.5	4.7	4.3	4.2	<LOD	3.8
C	5.9	<LOD	<LOD	<LOD	<LOD	3.0
C, cold	7.4	3.4	<LOD	<LOD	<LOD	4.2
C, hot	7.6	3.4	2.5	<LOD	<LOD	4.1
D	7.6	4.2	<LOD	<LOD	<LOD	4.3
E	ND	ND	ND	ND	ND	ND
E, cold	ND	ND	ND	ND	ND	ND
E, hot	ND	ND	ND	ND	ND	ND
F	6.9	3.3	<LOD	<LOD	<LOD	3.7
G	6.6	<LOD	<LOD	<LOD	<LOD	3.5

*Results were obtained by quantitative PCR targeting genes specific to the organism of interest. Results are shown as the log_{10} of gene copies per liter. LOD, limit of detection; ND, not determined because no sample was collected.

Appendix Table 10. Concentrations of various organisms in water samples collected from different locations within a community water system in May 2024*

Location	All bacteria	*Legionella* spp.	*L. pneumophila*	*L. pneumophila SG1*	*Acathamoeba*	*V. vermiformis*
Finished water	7.5	2.8	<LOD	<LOD	<LOD	4.2
A	7.4	3.5	<LOD	<LOD	<LOD	4.0
B	7.3	3.2	<LOD	<LOD	<LOD	4.0
B, cold	7.4	3.6	<LOD	2.5	<LOD	3.8
B, hot	8.2	4.4	3.1	3.0	<LOD	4.0
C	7.5	3.0	<LOD	<LOD	<LOD	4.2
C, cold	8.0	2.6	<LOD	<LOD	<LOD	4.0
C, hot	7.1	2.9	<LOD	<LOD	<LOD	3.6
D	7.7	3.4	<LOD	<LOD	<LOD	4.6
E	7.7	3.5	<LOD	<LOD	<LOD	4.7
E, cold	7.5	3.2	<LOD	<LOD	<LOD	4.5
E, hot	7.4	4.1	3.9	3.4	<LOD	4.4
F	7.7	4.0	<LOD	<LOD	<LOD	4.7
G	7.7	3.0	<LOD	<LOD	<LOD	3.7

*Results were obtained by quantitative PCR targeting genes specific to the organism of interest. Results are shown as the $\log_{10}$ of gene copies per liter. LOD, limit of detection.

Appendix Table 11. Concentrations of various organisms in water samples collected from different locations within a community water system in September 2024*

Location	All bacteria	*Legionella* spp.	*L. pneumophila*	*L. pneumophila SG1*	*Acathamoeba*	*V. vermiformis*
Finished water	5.2	<LOD*	<LOD	<LOD	<LOD	<LOD
A	5.4	<LOD	<LOD	<LOD	<LOD	<LOD
B	5.2	<LOD	<LOD	<LOD	<LOD	<LOD
B, cold	7.3	4.8	3.9	4.0	<LOD	<LOD
B, hot	8.8	8.2	8.2	8.1	<LOD	4.0
C	5.5	<LOD	<LOD	<LOD	<LOD	3.0
C, cold	5.0	3.4	<LOD	<LOD	<LOD	<LOD
C, hot	7.6	3.4	<LOD	<LOD	<LOD	5.0
D	6.1	3.0	<LOD	<LOD	<LOD	3.5
E	5.2	2.6	<LOD	<LOD	<LOD	<LOD
E, cold	6.5	3.6	<LOD	<LOD	<LOD	3.8
E, hot	6.1	3.5	2.6	<LOD	<LOD	3.2
F	5.3	3.4	<LOD	<LOD	<LOD	<LOD
G	6.3	2.4	<LOD	<LOD	<LOD	3.8

* Results were obtained by quantitative PCR targeting genes specific to the organism of interest. Results are shown as the $\log_{10}$ of gene copies per liter. LOD, Limit of detection.

Appendix Table 12. Concentrations of various organisms in water samples collected from different locations within a community water system in December 2024*

Location	All bacteria	*Legionella* spp.	*L. pneumophila*	*L. pneumophila SG1*	*Acathamoeba*	*V. vermiformis*
Finished water	5.8	< LOD*	< LOD	< LOD	< LOD	3.3
A	5.6	< LOD	< LOD	< LOD	< LOD	< LOD
B	5.9	< LOD	< LOD	< LOD	< LOD	3.1
B-cold	6.9	3.8	< LOD	< LOD	< LOD	3.4
B-hot	7.3	4.7	< LOD	< LOD	< LOD	3.7
C	5.2	< LOD	< LOD	< LOD	< LOD	< LOD
C-cold	5.2	2.4	< LOD	< LOD	< LOD	< LOD
C-hot	5.1	< LOD	< LOD	< LOD	< LOD	< LOD
D	6.2	2.6	< LOD	< LOD	< LOD	3.7
E	6.4	< LOD	< LOD	< LOD	< LOD	3.6
E-cold	5.8	3.6	< LOD	< LOD	< LOD	3.4
E-hot	NM†	NM	NM	NM	NM	NM
F	5.7	3.4	< LOD	< LOD	< LOD	3.1
G	7.3	2.4	< LOD	< LOD	< LOD	5.0

*Results were obtained by quantitative PCR targeting genes specific to the organism of interest. Results are shown as the $\log_{10}$ of gene copies per liter. LOD, Limit of detection; NM, Not measured because the sample was accidentally lost during processing.

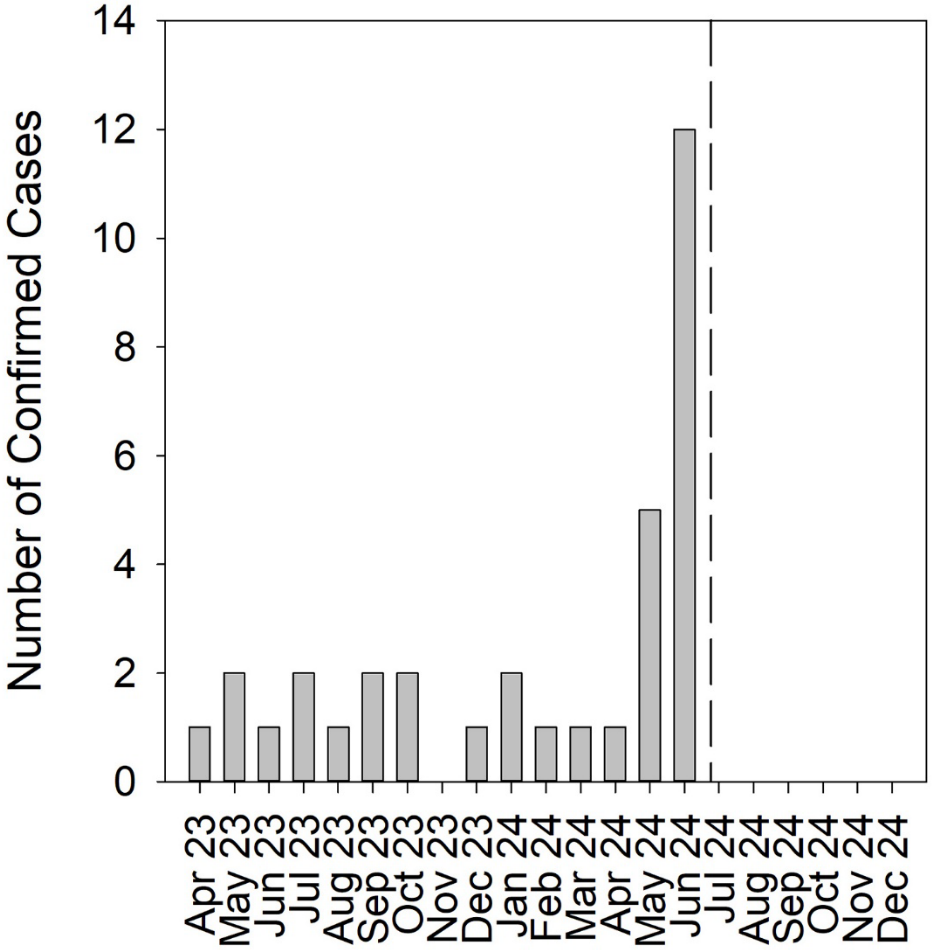

Appendix Figure. Number of confirmed cases of Legionnaires' disease in the community from April 2023 to December 2024. The dashed line represents the date of implementation of disinfection of the drinking water (June 2024). These publicly available data were obtained from the Minnesota Department of Health.
